# The cap-binding complex modulates ABA-responsive transcript splicing during germination in barley (*Hordeum vulgare*)

**DOI:** 10.1038/s41598-024-69373-9

**Published:** 2024-08-07

**Authors:** Ewa Sybilska, Anna Collin, Bahareh Sadat Haddadi, Luis A. J. Mur, Manfred Beckmann, Wenbin Guo, Craig G. Simpson, Agata Daszkowska-Golec

**Affiliations:** 1 Institute of Biology, Biotechnology and Environmental Protection, Faculty of Natural Sciences, University of Silesia in Katowice, Jagiellońska 28, 40-032 Katowice, Poland; 2 Department of Life Science, Aberystwyth University, Aberystwyth, UK; 3 Information and Computational Sciences, James Hutton Institute, Dundee, DD2 5DA Scotland, UK; 4 Cell and Molecular Sciences, James Hutton Institute, Dundee, DD2 5DA Scotland, UK

**Keywords:** ABA, Alternative splicing, Barley, Cap-binding complex, Embryo, Germination, Transcriptome, Abiotic, Plant molecular biology, Gene expression, Mutation, RNA splicing

## Abstract

To decipher the molecular bases governing seed germination, this study presents the pivotal role of the cap-binding complex (CBC), comprising CBP20 and CBP80, in modulating the inhibitory effects of abscisic acid (ABA) in barley. Using both single and double barley mutants in genes encoding the CBC, we revealed that the double mutant *hvcbp20.ab/hvcbp80.b* displays ABA insensitivity, in stark contrast to the hypersensitivity observed in single mutants during germination. Our comprehensive transcriptome and metabolome analysis not only identified significant alterations in gene expression and splicing patterns but also underscored the regulatory nexus among CBC, ABA, and brassinosteroid (BR) signaling pathways.

## Introduction

Seed germination encompasses a variety of processes that occur from imbibition to radicle emergence^1^. Monocot seeds comprise three main components: the embryo, the endosperm, and the protective seed coat^2^. Plant studies have revealed that embryos are transcriptionally and translationally active, and crucial for seed germination and plant establishment^3–9^. Spatial transcriptomic analysis of germinating barley grains showed high expression of genes involved in cell division, lipid metabolism and transfer, phytohormone signaling, and aquaporin in embryos. Additionally, transcription factors and translation-related genes are mainly expressed in embryonic tissues^9^. Interestingly, other studies have reported that more than 12,000 transcripts are stored in dry barley grains and activated during seed germination^4^.

Regulation of the seed germination process at the molecular level requires interaction between various phytohormones but demands a critical balance between abscisic acid (ABA) and gibberellic acid (GA)^10–12^. During dormancy regression, ABA degradation and GA biosynthesis is known to increase. High ABA content in seeds prolongs dormancy and delays seed germination. Thus, ABA and GA antagonistically control the seed germination process^13^. Plants have evolved complex mechanisms to adapt to the changing environmental conditions. One of these mechanisms is the inhibition of seed germination under stressful conditions. Various abiotic factors have been shown to initiate cellular ABA accumulation and the activation of ABA signaling^14–18^. The central player in ABA-mediated inhibition of seed germination is the basic leucine zipper (bZIP) transcription factor ABA INSENSITIVE5 (ABI5)^19,20^. In addition to ABA and GA, other phytohormones, including brassinosteroids (BRs), also control the seed germination process^21^. BRs antagonize ABA during seed germination by inhibiting the ABA signal transduction pathway^22^. This aligns with the observation that the BR signaling *bri1* (*brassinosteroid insensitive 1*) and *bin2-1* (*brassinosteroid-insensitive 2*) mutants and the BR biosynthesis *det2-1* (*deetiolated 2*) mutant, are hypersensitive to ABA during seed germination^13,23–26^. The critical repressor of BR signaling, BRASSINOSTEROID INSENSITIVE2 (BIN2), interacts with and phosphorylates SNF1-RELATED PROTEIN KINASE 2 (SnRK2) to promote its kinase activity^27^. Other studies have shown that, during seed germination, BIN2 stabilizes ABI5 through direct interaction and phosphorylation^22^.

Genes encoding crucial components of the ABA signaling pathway, the spliceosome and transcription factors undergo alternative splicing (AS), to generate various splice isoforms that fine-tune the seed germination process^28–33^. In AS multiple mRNA isoforms are produced from a single gene through differential intron and exon excision or retention in various combinations from pre-mRNA. Thus, AS regulates gene expression at the co-transcriptional level and increases protein and transcriptome diversity^34,35^.

The *CBP20* (*cap-binding protein 20*) and *CBP80* genes encode the small and large subunits of the 5' cap-binding CBC (cap-binding complex), respectively. CBC is involved in conserved processes related to RNA metabolism, including miRNA biogenesis and alternative splicing^36–38^. CBP20 and CBP80 are RNA-binding proteins (RBPs), and their interaction is essential for complex functionality and binding to RNA^39^. The CBC subunits were initially characterized in human HeLa cells, and subsequent studies identified their homologs in other organisms, including plants^40^. Interestingly, the aminoacid sequences of both CBC subunits are highly conserved across the species, including yeast, animals, and plants, confirming the regulatory importance of the CBC complex^41^. The Arabidopsis AtCBP20 protein shows 68% identity and 82% similarity to human CBP20 and approximately 53% identity and 77% similarity to its ortholog in yeasts. However, the larger AtCBP80 subunit is less conserved, showing 28% identity and 50% similarity to the human ortholog, as well as 22% identity and 42% similarity to the yeast protein^38^. In Arabidopsis, the CBP20 protein consists of a 138-amino acid core (N-terminal) region containing the RNA binding domain (RBD) with RNP2 and RNP1 motifs and a plant-specific 120-amino acid C-terminal tail. The RBD domain plays a crucial role in recognizing and binding mRNA cap structures, while the N-terminal part is essential for interacting with CBP80, stabilizing CBP20. The C-terminal region includes nuclear localization signals (NLS), facilitating the transport of the CBC complex from the cytoplasm to the cell nucleus. Unlike in animals, the plant CBP80 subunit lacks NLS signals and requires CBP20 for nuclear transport^41–43^. Computational modeling of CBP20 in barley revealed the presence of NLS in C-terminal region of protein^43^.

It is worth to note that mutants in genes encoding CBC subunits are viable and not very different phenotypically from the WT under optimal conditions. The *cbp20* and *cbp80* single mutants have been investigated in several plant species, including Arabidopsis, barley, and potatoes^43–49^. However, research on the *cbp20/cbp80* double mutant has been limited to molecular studies in mature Arabidopsis plants. This research discovered a profound role of the CBP80 subunit in AS regulation^38^.

Our study focused on barley (*Hordeum vulgare)* as an object, a diploid species (n = 7). The barley genome contains a single copy of the *HvCBP20* and *HvCBP80* genes. Single mutations in the *CBP20* and *CBP80* genes cause hypersensitivity to ABA during seed germination and confer drought tolerance to dicots and monocots^36–38,50^. To address the gap in knowledge regarding the function of the cap-binding complex (CBC) in the ABA signaling pathway during barley seed germination, we used TILLING to identify mutants *hvcbp20.ab* and *hvcbp80.b* in both *HvCBP20* and *HvCBP80* genes. We then crossed the single mutants to produce and identify the homozygous double mutant *hvcbp20.ab*/*hvcbp80.b*. We found that mutations in both CBC subunits resulted in a distinct ABA response that differed from that of single mutants. Through transcriptomic analyses, we identified differential gene expression and alternative splicing patterns. Notably, the *hvcbp20.ab*/*hvcbp80.b* double mutant exhibited altered AS regulation and significant changes in brassinosteroid signaling following ABA exposure which facilitated seed germination.

## Results

### Barley mutant in both CBC subunits is insensitive to ABA during seed germination

**Figure 1:**
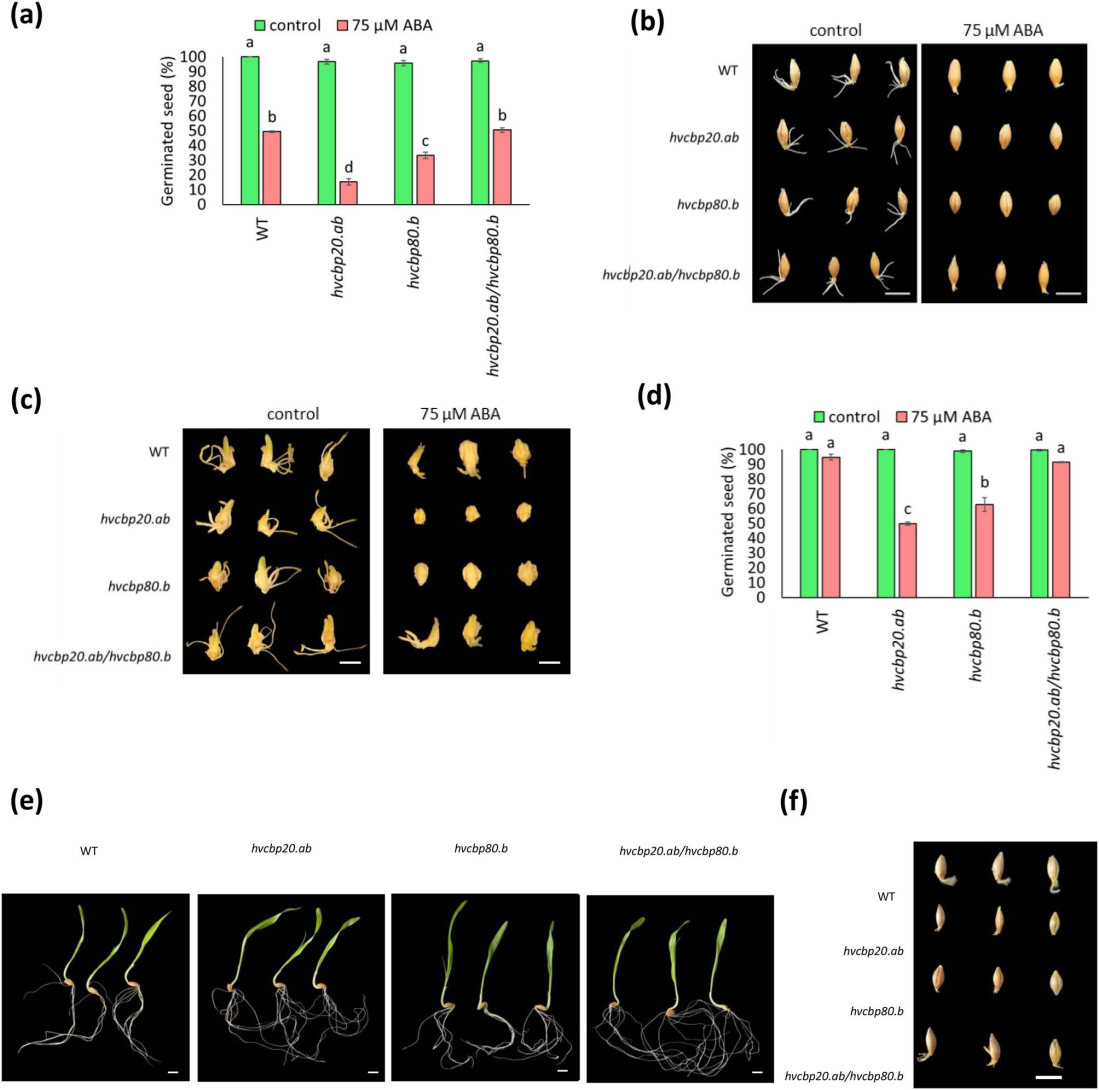
Germination of *hvcbp20.ab*, *hvcbp80.b*, *hvcbp20.ab/hvcbp80.b *and the WT in the presence of 75 µM ABA. (**a**) Seed germination percentage at 1 DAI. (**b**) Seed germination phenotypes at 1 DAI. Bar = 1 cm (**c**) Embryo germination phenotypes at 1 DAI. Bar = 3 mm. (**d**) Seed germination percentage at 7 DAI. (**e**) Seed germination phenotypes at 7 DAI in control conditions. (**f**) Seed germination phenotypes at 7 DAI in ABA presence. Bar = 1 cm DAI (day after imbibition). Statistical analyses were performed using one-way ANOVA (P ≤ 0.05) with post-hoc Tukey HSD (Honestly Significant Difference). Statistically significant differences (P ≤ 0.05) are marked by different lower-case letters.

In assessing ABA sensitivity at 1 DAI (Days After Imbibition) the *hvcbp20.ab*/*hvcbp80.b* (hereafter referred to as the double mutant) showed germination rates comparable to those of the wild-type (WT) when exposed to 75 µM ABA, in contrast to single mutants that exhibited significant germination inhibition (Fig. 1a–c). By 7 DAI, both the double mutant and WT seeds exceeded 90% germination, suggesting that the double mutation may counteract ABA-mediated inhibition, possibly by modulating the ABA-signaling pathway (Fig. 1d).

### Global analysis of *hvcbp20.ab/hvcbp80.b* embryos transcriptome in response to ABA

To better understand how the double mutant responded to ABA during seed germination, we performed a comparative analysis of the transcriptomes of all genotypes in the presence of ABA and under control conditions.

Under control conditions, there were only a few transcriptomic changes in all tested mutants compared to the wild type (WT). However, ABA treatment significantly increased the numbers of DEGs and DETs (Table 1). The double mutant in *CBP20* and *CBP80* genes showed the greatest transcriptional changes in response to ABA compared to the other genotypes. The numbers of differentially alternatively spliced (DAS) genes, differentially expressed transcripts (DETs), and differential transcript usage (DTU) was the highest in the double mutant. This number in *hvcbp20.ab*, *hvcbp80.b* and WT were reduced by over 30% compared to the amount of DAS, DET, DTU identified in the double mutant. Moreover, the double mutation significantly affected alternative splicing and transcript isoform formation. The intersection between DE/DAS genes and DET/DTU showed that 69 genes underwent AS only in the WT, 60 in *hvcbp20.ab*, 62 in *hvcbp80.b*, and 108 in the double mutant, corresponding to 68, 58, 60, and 71% of all DAS genes, respectively (Supplementary Fig. S2a). This suggests that DAS genes are mainly regulated at the alternative splicing level. Conversely, most DTU transcripts were produced by transcription in conjunction with AS, with DTU alone comprising 40% of the double mutant (Supplementary Fig. S2b).

**Table 1:** Number of DE/DAS genes and DE/DTU transcripts in different contrast groups.

Contrast	DE genes	DAS genes	DE transcripts	DTU transcripts
ABA.WT-control.WT	5533	102	8222	118
ABA.cbp20-control.cbp20	5657	103	8541	131
ABA.cbp80-control.cbp80	5914	103	9424	129
ABA.double-control.double	6309	152	11,733	196
control.cbp20-control.WT	19	2	16	2
control.cbp80-control.WT	85	5	46	3
control.double-control.WT	77	4	51	9

*DE* differential expression, *DAS* differential alternative splicing, *DTU* differentia transcript usage.

### Co-expression analysis reveals clusters of genes with a similar expression pattern in double mutant and WT in the presence of ABA

**Figure 2:**
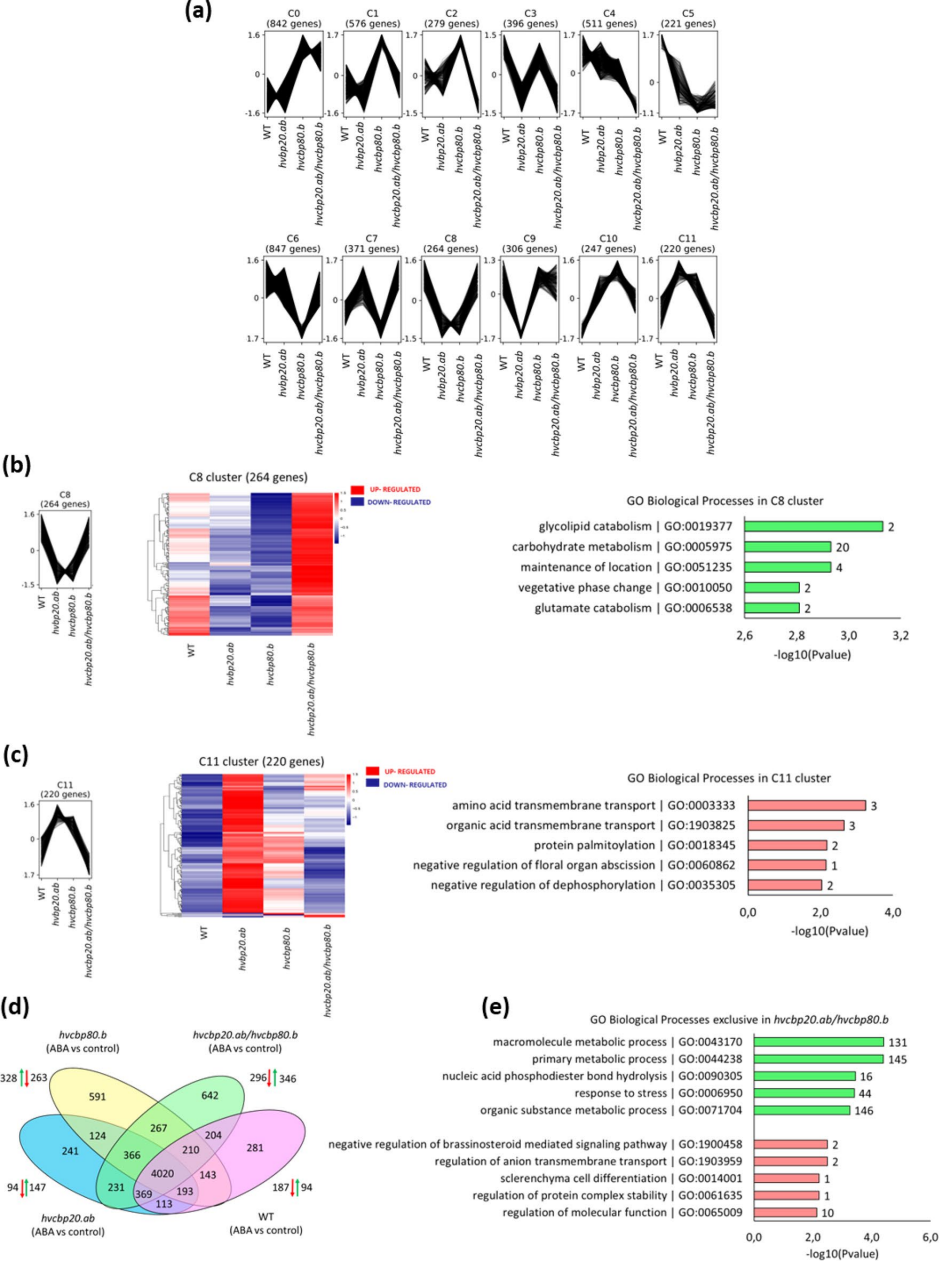
Global analysis of differentially expressed genes (DEG) of *hvcbp20.ab*, *hvcbp80.b*, *hvcbp20.ab/hvcbp80.b* and WT after 75 µM ABA treatment compared to control conditions. (**a**) Co-expressed clusters of DEGs in response to ABA. (**b**) Heatmap of DEGs and enriched GO biological processes in the C8 cluster. (**c**) Heatmap of DEGs and enriched GO biological processes in the C11 cluster. (**d**) Venn diagram of DEGs displaying up- and downregulated exclusively expressed genes in each contrast group. (**e**) Overrepresented GO biological processes among upregulated and downregulated exclusive DEGs in *hvcbp20.ab/hvcbp80.*b.

Co-expression analysis of DEGs (8070) identified in all genotypes tested in response to ABA during the seed germination revealed 12 gene clusters (Fig. 2a; Supplementary Data S1). The C8 and C11 clusters showed consistent expression patterns between the WT and the double mutant, contrasting with *hvcbp20.ab* and *hvcbp80.b*. In the C8 cluster, genes were upregulated in both the double mutant and WT, while being downregulated in the single mutants. On the contrary, the C11 cluster contained genes downregulated in the WT and double mutant but upregulated in *hvcbp20.ab* and *hvcbp80.b* single mutants. The Gene Ontology (GO) enrichment analysis of these clusters identified key biological processes that were prevalent in DEGs with similar expression patterns. In the C8 cluster, the glycolipid catabolic process (GO:0019377), carbohydrate metabolic process (GO:0005975), maintenance of location (GO:0051235), vegetative phase change (GO:0010050) and glutamate catabolic process (GO:0,006538) were the most significant GO terms (Fig. 2b). Whereas in the C11 cluster the top GO-BP were amino acid transmembrane transport (GO:0003333), organic acid transmembrane transport (GO:1903825), protein palmitoylation (GO:0,018,345), negative regulation of floral organ abscission (GO:0060862) and negative regulation of dephosphorylation (GO:0035305) (Fig. 2c).

### ABA-Induced expression of splicing factors and brassinosteroid signaling regulators unique to double mutant germination

We identified 642 DEGs specific to the double mutant. The single mutants *hvcbp20.ab* and *hvcbp80.b* displayed 241 and 591 unique DEGs, respectively, whereas the WT had 281 DEGs (Fig. 2d). Notably, the gene expression profile of the double mutant was significantly higher, with implications for the distinct from single mutants germination phenotype observed under ABA treatment. GO enrichment analysis of the upregulated gene set in the double mutant showed biological processes (BP) such as nucleic acid phosphodiester bond hydrolysis (GO:0090305)), suggesting an upregulation of splicing activity (Supplementary Data S2, Supplementary Data S3). Notably, six splicing factors have been identified, each with homologs in Arabidopsis, thereby providing a phylogenetic anchor for functional suggestions (Fig. 2e). These include CELL DIVISION CYCLE 5 (CDC5; BaRT2v18chr5HG272090), U2af small subunit A (U2AF35A; BaRT2v18chr5HG254110), SUPPRESSOR OF ABI3-5 (SUA; BaRT2v18chr3HG159600), embryo-defective 2016 (EMB2016; BaRT2v18chr7HG366170), and putative branchpoint-bridging protein-like (SF1; BaRT2v18chr2HG081650) (Supplementary Data S4). Conversely, genes implicated in the negative regulation of brassinosteroid signaling pathways (GO:1900458) were downregulated, highlighting a potential mechanistic intersection between ABA and BRs signaling, REMORIN (REM4.1; BaRT2v18chr2HG069620), BRI1 KINASE INHIBITOR 1 (BKI1; BaRT2v18chr5HG251390), and a previously uncharacterized gene (BaRT2v18chr5HG272750) (Supplementary Data S5). Collectively, these results delineate a distinct transcriptional landscape for the double mutant, with implications for its exclusive ABA-mediated germination phenotype, mediated in part by modulations in the splicing and brassinosteroid signaling pathways.

### Regulatory interplay between transcription factors, splicing factors, and brassinosteroid signaling inhibitors in double mutant

To elucidate the transcription factors (TFs) that potentially orchestrate the regulation of gene expression in response to ABA, we analyzed DEGs after ABA treatment across all genotypes (Supplementary Data S6). This yielded a list of 24 TFs in the double mutant, which starkly contrasts with the 6, 21, and 9 TFs found in *hvcbp20.ab*, *hvcbp80.b*, and WT, respectively. Notably, most TFs identified in the double mutant were upregulated, corroborating the enhanced transcriptional activity observed in these embryos upon ABA exposure.

The TFs encompassed 12 distinct transcription domain families (Supplementary Table 1), suggesting diverse regulatory capacities for modulating ABA responses during the seed germination stage. We next focused on prediction of transcription factor-binding sites (TFBSs) within promoters of DEGs specific to the double mutant. In response to ABA, six TFs were associated with 1178 TFBSs among the 392 DEGs specific to the double mutant suggesting potential regulatory complexity (Supplementary Data S7). A subset of these TFs was identified as having binding sites within DE genes encoding critical splicing factors. One TFBS was within the promoter of the negative regulator of BR signaling (REM4.1; BaRT2v18chr2HG069620) and one TFBS within *serine/threonine-protein kinase SAPK10* (SAPK10; BaRT2v18chr7HG385230) (Supplementary Data S8). These results suggest that the identified TFs may modulate the expression levels of genes relevant to the seed germination phenotype of double mutants in the presence of ABA, which may be CBC-dependent.

### Alternative splicing is impaired in germinating embryos of hvcbp20.ab/hvcbp80.b in the presence of ABA

**Figure 3:**
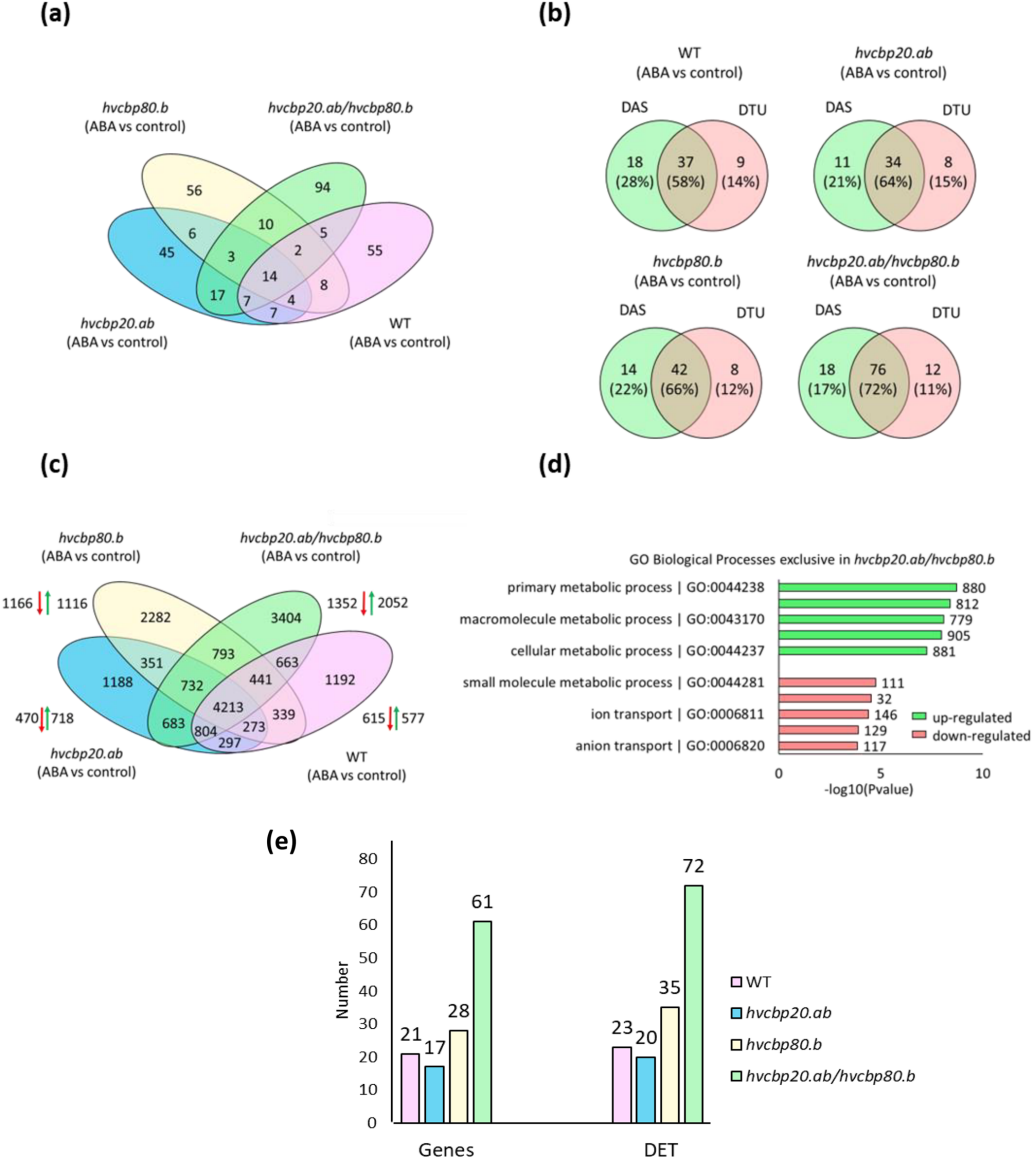
Global analysis of genes with differential alternative splicing (DAS) genes and differentially expressed transcripts (DET) in *hvcbp20.ab*, *hvcbp80.b*, *hvcbp20.ab/hvcbp80.b* and WT after 75 µM ABA treatment compared to control conditions. (**a**) Venn diagram of DAS genes in each contrast group. (**b**) Venn diagrams displaying the amount of exclusive DAS genes with DTU transcripts. (**c**) Venn diagram of DETs displaying up- and downregulated exclusively expressed transcripts in each genotype. (**d**) Overrepresented GO biological processes among upregulated and downregulated exclusive DETs in *hvcbp20.ab/hvcbp80.b*. (**e**) The number of genes encoding transcripts of splicing factors (SF) with the corresponding number of transcripts of SFs among exclusive DET in each genotype.

To compare changes in the alternative splicing pattern in the double mutant, *hvcbp20.ab*, *hvcbp80.b* and the WT in response to ABA, the sets of DAS genes were compared (Fig. 3a). Most genes that underwent splicing were uniquely spliced in each genotype. However, the largest number of DAS genes was identified in the double mutant (Supplementary Data S9). To identify the specific transcripts contributing to the classification of a gene as DAS, we performed a differential transcript usage (DTU) analysis. This process identified individual transcripts that exhibited significant differences relative to the other transcripts from the same gene. In the double mutant, 72% of exclusive DAS genes contained DTU transcripts. For *hvcbp20.ab* and *hvcbp80.b*, the DAS&DTU amounted to 64% and 66%, respectively. In contrast, the lowest number (58%) of exclusive DAS genes with corresponding exclusive DTU was identified in the WT (Fig. 3b; Supplementary Data S10). Hence, these data indicate that the double mutation exerts a pronounced effect on AS, resulting in a significant number of unique DTUs that align with the elevated number of DAS genes.

### Transcripts of splicing factors accumulate during seed germination in double CBC mutant in the presence of ABA

To reveal significant differences in transcript abundance between the double mutant and the single *hvcbp20.ab* and *hvcbp80.b* mutants, together with the WT in response to ABA, analyses were performed at the transcript level by examining the DET. The highest number of DETs (3404) was exclusively expressed in the double mutant. In the other genotypes, 1188 DETs were exclusive to *hvcbp20.ab*, 2282 to *hvcbp80.b* and 1192 to the WT (Fig. 3c). As the CBC complex is involved in RNA metabolism, including AS, the analysis of isoforms in *hvcbp20.ab/hvcbp80.b* will suggest the roles of the CBC in the response to ABA (Supplementary Fig. S3). The double mutant exhibited the highest number of events across most features of AS transcripts, particularly the premature termination codons (PTC) and non-sense mediated decay (NMD) categories, suggesting a possible interaction between the *CBP20* and *CBP80* genes in response to ABA treatment. GO enrichment analysis showed that genes encoding exclusively upregulated transcripts (DET) in double mutant are involved in GO 'biological process' (GO-BP) that involve metabolism and genes downregulated involve transcripts engaged in small molecule metabolic processes and anion and ion transport (Fig. 3d; Supplementary Data S11).

Furthermore, to quantify the modifications in the AS machinery attributable to the double mutation, transcripts linked to RNA splicing (GO:0008380) were catalogued among the uniquely expressed DETs in the double mutant and compared to the single mutants and WT. This led to the identification of 61 genes that accounted for 72 unique DETs in the double mutant, a notable enrichment that may signify substantial reprogramming of the splicing landscape, indicative of an adaptive response to ABA (Fig. 3e; Supplementary Data S12).

### Predicted interactions of CBC complex subunits with RNA metabolism and splicing factors

**Figure 4:**
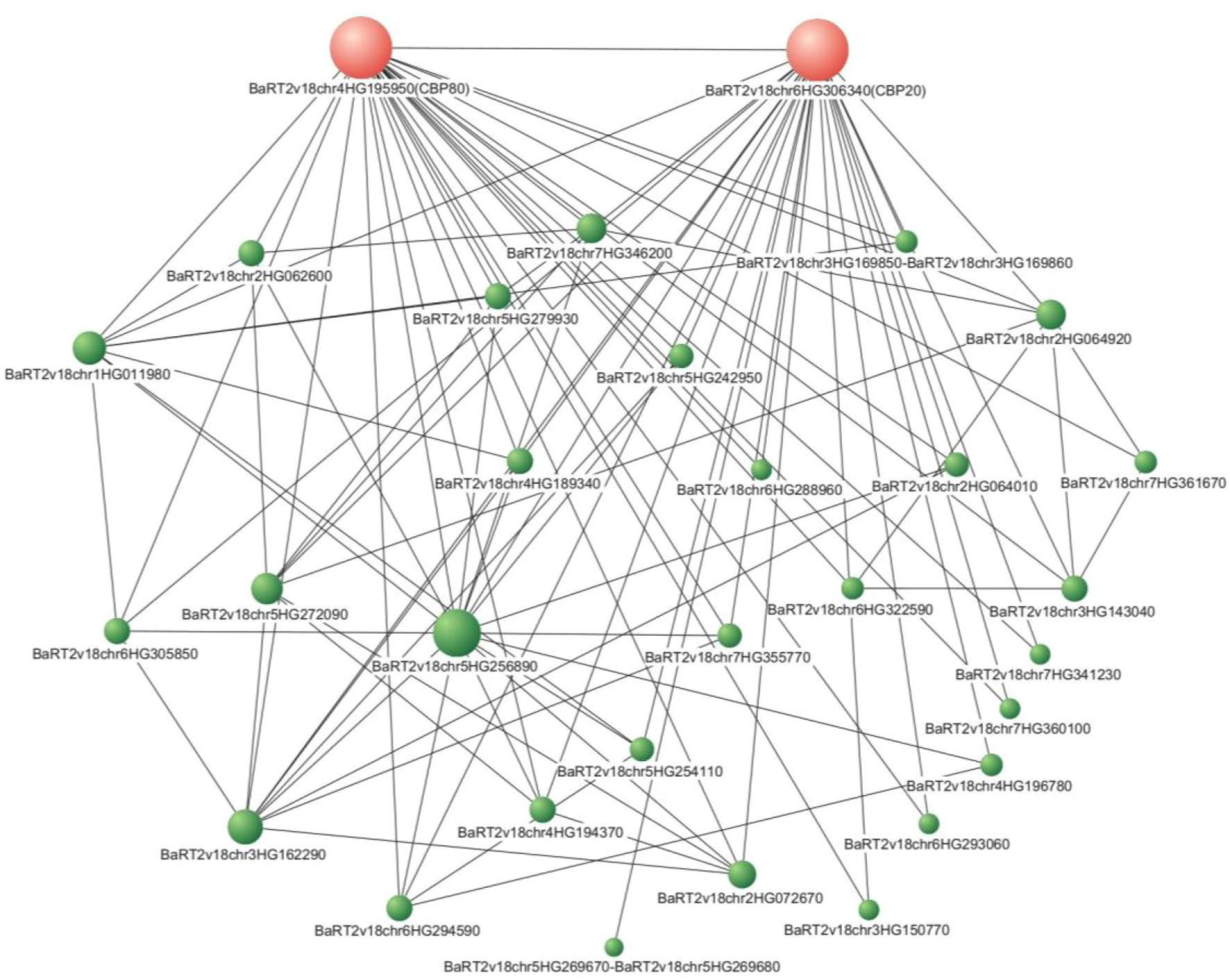
Network of protein–protein in silico physical interaction with CBP20 (red) and CBP80 (red) among differentially expressed genes (DEG) and genes with differential alternative splicing (DAS) in *hvcbp20.ab*, *hvcbp80.b*, *hvcbp20.ab/hvcbp80.b* and WT after 75 µM ABA treatment compared to control conditions.

To test whether the proteins encoded by the DEGs and DAS genes, identified specifically for *hvcbp20.ab/hvcbp80.b* embryos in the presence of ABA, demonstrated direct physical interactions with the CBC subunits, a putative model of protein–protein interaction (PPi) was devised using the STRING database (Fig. 4, Supplementary Data S13). Utilizing CBP20 and CBP80 as seeds in this analysis, our search revealed 28 proteins that exhibited physical interactions with CBC subunits, as corroborated by the experimental data deposited in STRINGdb. A breakdown of these interactions revealed that 20 proteins interacted with both CBP20 and CBP80, while four interacted only with the CBP20 subunit, and another four with CBP80 (Supplementary Data S14). Intriguingly, within this interactome, seven genes were classified as DEGs and two as DAS, identified specifically in *hvcbp20.ab/hvcbp80.b*. These genes predominantly function in RNA processing and splicing, thereby highlighting the interconnectedness between these processes and the CBC complex. Notably, these DEGs are genes encoding SF, including arginine/serine-rich 4 (BaRT2v18chr1HG011980), which interacts with both CBC subunits. Moreover, two splicing factors that interact solely with CBP20 are noteworthy: the splicing factor U2af small subunit A (U2AF35A; BaRT2v18chr5HG254110) and the CELL DIVISION CYCLE 5-like protein (CDC5; BaRT2v18chr5HG272090) as detailed in Supplementary Data S14. These results showed an association between the CBC complex and other splicing and RNA processing factors, confirming its involvement in the regulation of seed germination via AS. The proteins encoded by these genes might act as CBC-dependent regulators of seed germination in the presence of ABA.

### Brassinosteroid biosynthesis may be increased in *hvcbp20.ab/hvcbp80.b* mutant in the presence of ABA

**Figure 5:**
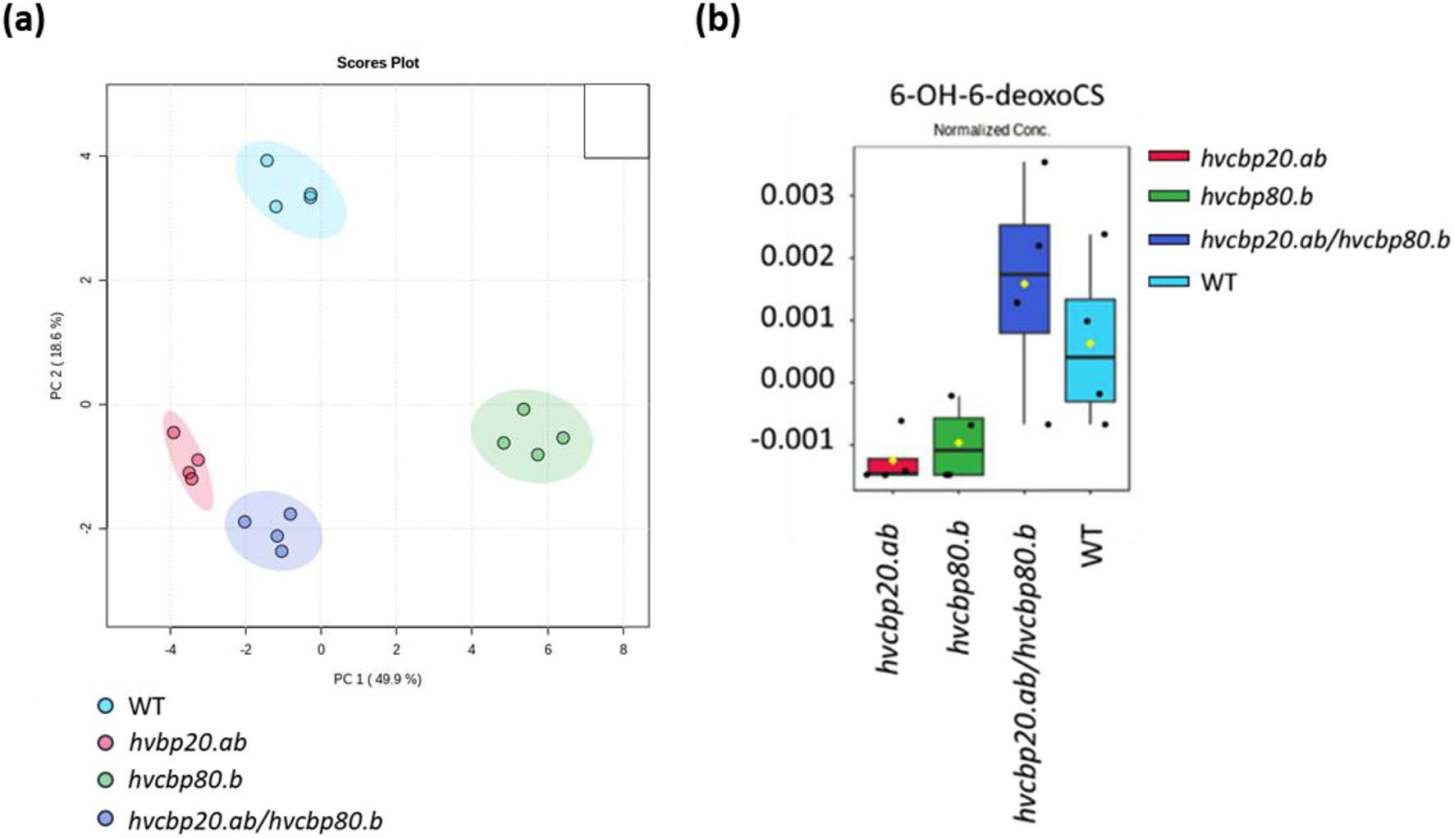
Metabolome profiling analysis of germinating embryos of *hvcbp20.ab*, *hvcbp80.b*, *hvcbp20.ab/hvcbp80.b* and WT after 75 µM ABA treatment. (**a**) Principal component analysis (PCA) score plot of the metabolic profiles. (**b**) Endogenous 6-alpha-hydroxy-6-deoxycastasterone (6-OH-6-deoxoCS) content.

Alterations in BR signaling were observed in the double mutant through transcriptome profiling. Gene ontology enrichment analysis of genes specifically upregulated in double mutant demonstrated that the ‘negative regulation of BR signaling’ was overrepresented as the top Biological Process of GO categories. Additionally, genes encoding negative regulators of BR signaling were exclusively downregulated in the double mutant in response to ABA. To determine whether the ABA-insensitive phenotype also involves changes in BR biosynthesis, we analyzed distinctive the metabolomic profiles of germinating embryos from all genotypes under ABA treatment (Fig. 5a). The metabolomes were interrogated, and the sources variation were identified. Focusing on those sources of variation linked to hormones, this revealed significantly increased levels of 6-alpha-hydroxy-6-deoxycastasterone, a brassinolide biosynthesis intermediate, in the double mutant when compared to the WT and other genotypes. This upregulation aligns with the observed germination patterns, in which the double mutant demonstrated an enhanced germination rate, suggesting a growth-promoting effect and diminished ABA sensitivity (Fig. 5b).

### Towards Sebastian RTD: capturing genotype-specific transcriptome variations

To assess whether the BaRTv2.18 transcriptome^51^, based on the Barke genotype, potentially overlooked transcriptomic data specific to the Sebastian genotype, adhered to the BaRTv2.18 assembly process the Reference Transcriptomic Dataset (RTD) named BarkeRTD was generated. This included assembling reference transcript datasets (RTDs) from Illumina RNA-seq short reads (RNAseq RTD) and PacBio Iso-seq long reads (Isoseq RTD comprised eight samples from this study representing each genotype under control and ABA), by using the same Barke reference genome as outlined by Ref.^51^. RNAseq RTD and Isoseq RTD were merged to produce a unified BarkeRTD. Comparative analysis indicated that BaRTv2.18 exhibits slightly higher genome coverage than BarkeRTD. This was due to the mapping of the Sebastian sequencing data to the Barke reference genome, potentially leading to the omission of Sebastian-specific genes^52^. However, BarkeRTD demonstrated notable augmentation, featuring approximately 5000 additional genes and 9000 more transcripts (Supplementary Table 2).

**Figure 6:**
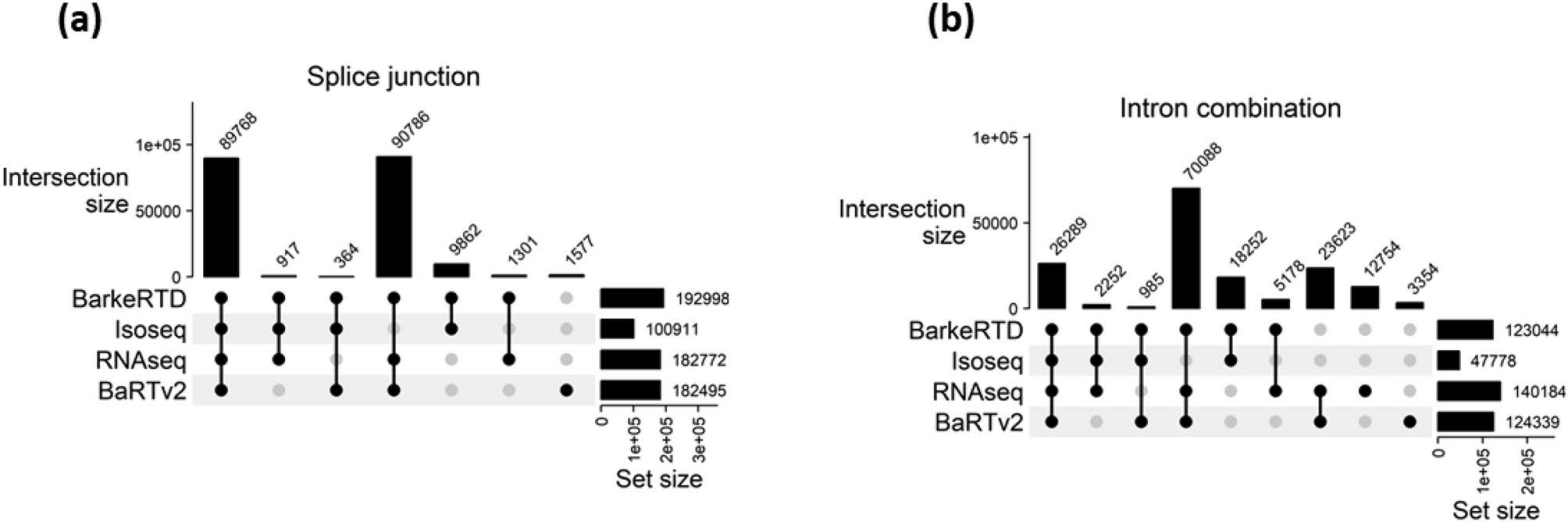
Comparisons of BarkeRTD, Isoseq RTD, RNAseq RTD and BaRTv2.18 on (**a**) splice junctions and (**b**) intron combinations of multi-exon transcripts. Splice junctions or Intron combinations shared by multiple transcripts were only counted once.

The integration of RNAseq and Isoseq RTDs resulted in a substantial contribution, introducing over 12,000 novel splice junctions and 25,700 novel intron combinations to enrich the BarkeRTD, surpassing the content available in BaRTv2.18 (Fig. 6a,b). It is worth to note that none of the tissues used to produce BaRTv2.18 represent germinating barley embryos and we therefore prepared an RTD, also against Barke, that utilised our sequence data to provide sequence information that may be additional to BaRTv2.18. Our commitment to enhancing the dataset continues with the ongoing generation of new Iso-seq data. We are optimistic about achieving an even more comprehensive and refined Sebastian RTD using a Sebastian-based reference genome, coupled with additional sequencing data in the future.

## Discussion

Mutations in *CBP20* and *CBP80* confer hypersensitivity to ABA during seed germination in Arabidopsis and barley (*Hordeum vulgare*)^43–48^. Therefore, we expected the same ABA phenotype for the *hvcbp20.ab/hvcbp80.b* double mutant. However, our results demonstrated that the germination rate of the double mutant was comparable to that of the wild type (WT) in the presence of ABA, which is in striking contrast to the inhibition of germination in single mutants.

In Arabidopsis, the *cos1 coi1-2* double mutant exhibits resistance to pathogens and a deviation in senescence patterns^53^, mirroring the phenomenon described in this study in the double mutant *hvcbp20.ab/ hvcbp80.b*. A reversal of the effect of the mutation in the *da3-1* single mutant, which leads to curly leaves and increased organ sizes, was observed in *sud1-1 da3-1* and *sud2-1 da3-1* double mutants^54^. Similarly, the growth of *bri1–5/bri1–1D*, *bri1–5/brs1–1D* and *bri1–5/bak1–1D* double mutants is partially restored compared to the dwarf *bri1–5* single mutant with the mutation in the *BRASSINOSTEROID-INSENSITIVE 1* (*BRI1*) gene from the BR signal transduction pathway^55^.

In all these examples, the double mutants complemented the genetic relationship, presenting a reversion to a WT-like phenotype that was not observed in the single mutants. This could imply a buffering effect or compensatory genetic interaction, where the confluence of two mutations mitigates the sensitivity observed in the single mutants. We cannot rule out a scenario in which the two subunits of the CBC can interact when both are mutated, while the interaction between mutated CBP20 and WT CBP80 or vice versa is not efficient. However, further experimental validation is required to confirm this. The double mutant insensitivity to ABA also suggests compensatory transcriptional reprogramming, possibly through altered expression or function of key genes within the ABA response network. This hypothesis was supported by the elevated transcriptional activity of the double mutant. Comparative transcriptome analysis revealed that the double mutant exhibited the most significant changes after ABA treatment, with a 30% increase in differentially expressed genes and transcripts relative to other genotypes. The prominence of differentially alternatively spliced genes in the double mutant suggests a reconfiguration of the splicing landscape, potentially contributing to the observed phenotype. The action of the CBC complex is notably more important in response to stress or ABA, evidenced by the minimal transcriptomic changes observed in the studied mutants compared to the WT under control conditions. These findings align with previous studies on the CBC complex in barley and Arabidopsis under stress conditions^43,56^.

In plants, the involvement of the CBC complex in the constitutive and alternative splicing of pre-mRNA has been documented, where the CBC, together with the zinc finger protein SERRATE (SE), acts as a platform for interaction with other splicing factors^37,38^. Thus, we hypothesized that the role of CBC in seed germination may be splice-dependent. RNA-seq analysis of the embryo transcriptome in response to ABA revealed that genes and transcripts encoding splicing factors were specifically downregulated in the double mutant compared to *hvcbp20.ab, hvcbp80.b,* and WT. Among the DEG, we identified six genes encoding splicing factors that were upregulated only in the double mutant*s* in the presence of ABA. One of them was *CELL DIVISION CYCLE 5* (*CDC5*) which encodes a Myb-related protein that is a subunit of the nuclear MOS4-ASSOCIATED COMPLEX (MAC) in Arabidopsis, important for the regulation of plant innate immunity^57^. Interestingly, CDC5 interacts with SE and DICER-LIKE 1 (DLC1) during miRNA biogenesis and mediates alternative splicing of RESISTANCE TO RALSTONIA SOLANACEARUM 1 (RRS1) and RPS4^58–60^. The interactome analysis revealed a physical interaction between CBP20 and CDC5. The next step is to validate the relationship between the CBC complex, CDC5, and SE in AS regulation during seed germination. The other identified splicing factor is the U2 auxiliary factor (U2AF), which is a part of the core of the spliceosome^61^. U2AF is a heterodimer composed of the U2AF35 and U2AF65 subunits. U2AF35 binds to the 3′ splice site, whereas U2AF65 binds to the polypyrimidine tract (PPT) of nascent pre-mRNA^62^. Moreover, U2AF35 undergoes alternative splicing to form two isoforms, U2AF35a and U2AF35b, which interact with U2AF65^63^. Other studies in Arabidopsis have shown that U2AF65a and U2AF65b interact with SPLICING FACTOR 1 (SF1)^64,65^. SF1 acts as a splicing enhancer and is a member of the serine-arginine-rich (SR) protein family that facilitates spliceosome assembly^64,66–69^. Several studies have revealed that SR proteins regulate plant responses to environmental stress via AS^70–73^.

Our study suggests a physical interaction between CBP20 and U2AF35a (BaRT2v18chr5HG254110) based on interactome analysis and identified increased expression of the putative branchpoint-bridging protein-like (SF1; BaRT2v18chr2HG081650), suggesting its involvement in the regulation of seed germination. Another factor from the SR family identified in our study that probably interacts with both CBC subunits is an arginine/serine-rich 4 (BaRT2v18chr1HG011980) a homolog of AtRS2Z33. In Arabidopsis, ectopic expression of *AtRS2Z33* affects the AS of other genes, as well as the AS of its pre-mRNAs, generating splice variants containing premature termination codons (PTC), which are then directed into the nonsense-mediated decay (NMD) pathway. It ensures an important post-transcriptional mechanism linking AS with NMD and regulates cellular protein levels in the cells^74,75^. Taken together, the altered expression of these splicing factors exclusively in *hvcbp20.ab/hvcbp80.b* suggests their potential dependency on the entire CBC complex. In addition, the potential interaction between the subunits of the CBC complex and the homologs of AtCDC5, AtU2AF35b/AtU2AF35a, and AtRS2Z33 suggests their common role during germination in the presence of ABA, which should be further investigated. We speculate that the 1.5-fold higher amount of DAS in *hvcbp20.ab/hvcbp80.b* compared to *hvcbp20.ab*, *hvcbp80.b,* and WT is related to the altered expression patterns of the identified SFs.

Raczynska et al.^38^ investigated young Arabidopsis seedlings and suggested that the entire CBC complex is involved in alternative splicing, with an emphasis on the first intron of the transcript. The CBP80 subunit plays a substantial role in alternative splicing, as demonstrated by the fact that the *Atcbp80/abh1* mutant and the double mutant displayed significantly more common changes in alternative splicing levels than the *Atcbp20* mutant and the double mutant. In our study, we did not observe such a relationship because there were 17 common DAS genes for *hvcbp20.ab* and the double mutant and 10 for *hvcbp80.b* and the double mutant. Most DAS genes (94 DAS) were exclusive to *hvcbp20.ab/hvcbp80.b*, of which 76 DAS (81%) contained DTU transcripts. This indicated the importance of the entire barley CBC complex interaction in AS regulation during the seed germination stage.

Our approach, focusing on gene and transcript level expression changes, aimed to comprehensively investigate how the CBC influences alternative splicing dynamics, resulting in the generation of diverse transcript isoforms. This allowed us to observe the effect of CBC on AS not only at the gene level but also directly at the transcript level. Mutations in both CBC subunits led to a significantly increased accumulation of SF transcripts in the *hvcbp20.ab/hvcbp80.b* mutant. Among the 72 identified DET in SF, the presence of isoforms encoding homologs of AtSE, AtPRP4KA, AtPININ, and AtSR45 were particularly interesting. We observed the accumulation of two SF-coding transcripts (BaRT2v18chr7HG374470.5, BaRT2v18chr7HG374470.3), specifically in *hvcbp20.ab/hvcbp80.b*. Recent studies have shown that SE is also regulated by phosphorylation by the pre-mRNA PROCESSING FACTOR 4 KINASE A (PRP4KA), affecting its protein activity and proper accumulation in cells^76^. The *PRP4KA* transcript (BaRT2v18chr5HG220870.3) was also strongly upregulated in *hvcbp20.ab/hvcbp80.b* embryos in the presence of ABA. These results suggest a potential relationship among CBC, SE, and PRP4KA in fine-tuning germination in response to ABA, which requires further evaluation. We detected increased expression of two transcripts of the *AtSR45* homolog (BaRT2v18chr3HG170170.2 and BaRT2v18chr3HG170170.8), which is a highly conserved RNA-binding protein. SR45 interacts with the ABA-dependent splicing factors SNW/SKI-INTERACTING PROTEIN (SKIP) and SUA, and approximately 30% of the transcripts that bind to SR45 are involved in the ABA-signaling pathway^77,78^. Moreover, SR45 interacts with other SFs, including U2AF35b and its paralogs U2AF35, RSZ21, and SR34a, which were upregulated in our study (BaRT2v18chr3HG143420.7, BaRT2v18chr6HG321740.4, BaRT2v18chr6HG321740.5, and BaRT2v18chr2HG059400.6)^79–82^. This suggests the existence of an ABA-dependent interaction network that facilitates spliceosome assembly at the seed germination stage, in which the CBC complex is involved.

The upregulation of specific transcription factors in the double mutant further suggests a broad regulatory capacity, with the potential to reshape the ABA response during the germination stage. The highest number of TFs identified in *hvcbp20.ab/hvcbp80.b* corresponded to the largest changes in DEG and DET levels in this mutant after ABA treatment. Five of the exclusively expressed TFs were particularly interesting because they potentially bind to the promoters of the splicing factors *AtCDC5*, *AtEMB2016*, *AtSF1*, *AtRS2Z33*, *AtU2AF35b*/*AtU2AF35a* and *AtSUA*. These TFs are likely to also regulate *AtREM4.1* and the SAPK10 homolog of *AtSnRK2*s is important for ABA and BRs signaling.

ABA and BR antagonistically regulate various plant developmental processes, including seed germination^23,83^. A particularly interesting finding was that the ‘negative regulation of brassinosteroids’ was within the most significant biological process (BP) in the GO category in germinating embryos of double mutant in the presence of ABA. Interestingly, negative regulators of BR signaling were specifically downregulated in the double mutant (Supplementary Data S5). We detected reduced expression of *REMORIN* (REM4.1; BaRT2v18chr2HG069620) and *BRI1 KINASE INHIBITOR 1* (BKI1; BaRT2v18chr 5HG251390). In Arabidopsis, insertional mutants in *REM4.1* exhibit strong insensitivity to ABA during seed germination^84^. This mutant was even more insensitive than the *abi1-1 (abscisic acid-insensitive 1–1)* mutant^85^. When the level of ABA in the cell increases, *OsREM4.1* is repressed by OsbZIP23 (OsABF4). Subsequently, OsREM4.1 inhibits BR signaling by disrupting the complex formation of OsBRI1 and OsSERK1^86^. In our study, we did not identify a homolog of the bZIP23 transcription factor among exclusive DEGs in *hvcbp20.ab/hvcbp80.b*. However, we observed reduced expression of the transcription factor TGACG MOTIF-BINDING FACTOR 6 (TGA6; BaRT2v18chr1HG033440), which belongs to the basic leucine zipper (bZIP) gene family. TGA6 contains a TFBS in the promoter sequence of *REM4.1*, thus potentially regulates *REM4.1*.

BRI1 INHIBITING KINASE 1 (BKI1), such as REM4.1, interferes with the formation of the BRI1 complex with BRASSINOSTEROID RECEPTOR INSUSPENSIVE KINASE 1 (BAK1)^87–90^. Therefore, it is possible that REM4.1 and BKI1 act as independent negative regulators of BR signaling^86^.

**Figure 7:**
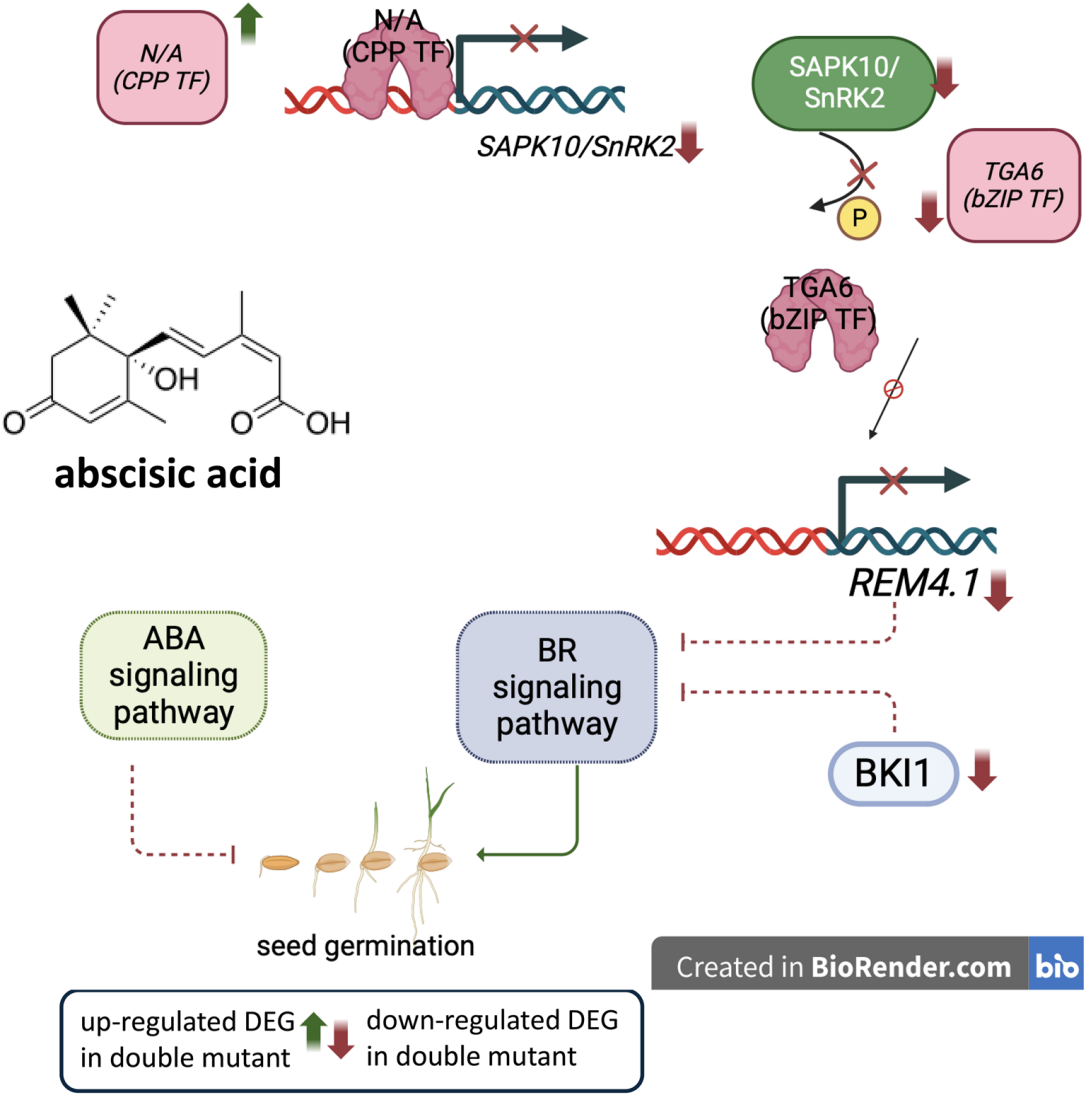
Hypothetical model explaining the seed germination phenotype of the *hvcbp20.ab/hvcbp80.b* in the presence of ABA linked with BR signaling. In the presence of ABA in the *hvcbp20.ab/hvcbp80.b* double mutant, the unknown transcription factor from the CPP-family is exclusively upregulated. This transcription factor potentially binds *SAPK10* and inhibits its expression. It leads to reduced phosphorylation of the TGA6 transcription factor by SAPK10 and reduced expression of the *REM4.1* gene encoding a negative regulator of BR signaling. Additionally, in *hvcbp20.ab/hvcbp80.b,* in response to ABA, the *BKI1* gene encoding the negative regulator of the BR signaling pathway is exclusively downregulated. All this together causes the seed germination process in the *hvcbp20.ab/hvcbp80.b* double mutant to be inhibited in the presence of ABA. The dashed lines represent possible interactions. The solid lines represent known interactions. The green color indicates exclusively differentially upregulated genes in *hvcbp20.ab/hvcbp80.b* in response to ABA. The red indicates exclusively differentially downregulated genes in *hvcbp20.ab/hvcbp80.b* in response to ABA. P- phosphorylation. Illustration created with BioRender.

In addition, among the specifically downregulated genes in the presence of ABA in *hvcbp20.ab/hvcbp80.b*, we observed the gene encoding the serine/threonine-protein kinase SAPK10 (SAPK10; BaRT2v18chr7HG385230), which is highly homologous to SnRK2s in Arabidopsis. ABA induces *OsSAPK10* and its overexpression confers hypersensitivity to ABA^91^. In plants, SnRK2 kinases physically bind and phosphorylate ABF to transmit ABA signals^92^. Moreover, our interactome analyses showed that upregulated transcription of TF from the CYSTEINE-RICH POLYCOMB-LIKE PROTEIN (CPP) family (BaRT2v18chr1HG048310) that potentially binds to SAPK10. To understand the regulatory mechanism, we need to confirm whether the transcription factor CPP interacts with *SAPK10 in planta* and whether SAPK10 adds a phosphate group to TGA6, further influencing *REM4.1*. Additionally, the metabolome results showed increased concentrations of castasterone, which is a precursor to brassinolide (BL), the most active BR^93,94^, in germinating embryos of *hvcbp20.ab/hvcbp80.b* and WT plants in the presence of ABA. Considering these results, we speculate that the downregulation of *REM4.1* and *BKI1* genes from the BR signaling pathway in the presence of ABA, together with potentially increased BR biosynthesis, resulted in the promotion of seed germination in the double *hvcbp20.ab/hvcbp80.b* mutant. Our findings demonstrate that the disruption of both CBC subunits potentially impairs the crosstalk between ABA and BR signaling and may induce BR biosynthesis. However, further research is required to understand the biological and genetic bases of these observations. We propose a hypothetical model of action for the identified factors in *hvcbp20.ab/hvcbp80.b* explaining its seed germination phenotype in the presence of ABA linked to signaling (Fig. 7).

In conclusion, our investigation demonstrated that the barley double mutant *hvcbp20.ab/hvcbp80.b* exhibits resistance to ABA during seed germination, suggesting that simultaneous mutations in the CBC subunits can mitigate ABA sensitivity through genetic interactions. Our detailed transcriptomic analysis of ABA responses highlights significant transcriptional reorganization, marked by shifts in gene expression, alternative splicing, and transcript isoform variation. The distinct phenotypes of the double mutant likely arise from the activation of specific gene and transcript sets depending on both CBC subunits. This study is the first to establish the crucial role of CBC in ABA-mediated seed germination in barley, particularly through its influence on alternative splicing and its interplay with the ABA and BR signaling pathways. Moreover, we have identified several key components of signaling pathways dependent on CBC, and thus warrant further investigation, which paves the way for future studies to comprehensively decode CBC function in ABA signaling during germination.

## Materials and methods

### Plant material

We used grains and embryos of barley TILLING mutants: the *hvcbp20.ab,* in the *CBP20* gene (*CAP-BINDING PROTEIN 20*; BaRT2v18chr6HG306340; Refs.^43,95^), and *hvcbp80.b*, in the *CBP80* gene (*CAP-BINDING PROTEIN 80*; BaRT2v18chr4HG195950), single mutants, the *hvcbp20.ab*/*hvcbp80.b* double mutant, and the 'Sebastian' wild-type (WT), all sourced from the *Hor*TILLUS population^96^. Single mutants were identified through TILLING in the M_2_ generation (then backcrossed with WT to clean the genetic background), and the double mutant was identified via genotyping the F_2_ progeny following crossbreeding of the single mutants (Supplementary Fig. S1). Mutations in the *CBP20* and *CBP80* genes in *hvcbp20.ab/hvcbp80.b* double mutant were confirmed using Sanger sequencing in three biological replicates in two consecutive generations of double mutant after self-pollination series.

### Seed germination assay in the presence of 75 µM ABA, embryos isolation and RNA extraction

Seeds were sterilized in 20% sodium hypochlorite solution for 20 min and washed with sterilized water for 5 min. They were then placed in Petri dishes with three layers of filters and treated with either sterilized water or sterilized water containing 75 μM ABA (cis–trans-abscisic acid; catalog no. 862169; Sigma-Aldrich). The seeds were stratified for 4 d at 4 °C and then transferred to a growth chamber. Germination was defined as the visible emergence of the radicle through the seed coat and was assessed on 1 and 7 DAI (days after imbibition). The assay was performed in three biological replicates, each comprising 30 seeds of each genotype per petri dish. At 1 DAI, the embryos were isolated from the endosperm using a sharp scalpel blade, placed in microcentrifuge tubes (Eppendorf) containing RNAlater reagent, and stored at 4 °C until RNA isolation. RNA from isolated germinating embryos at 1 DAI was extracted using the mirVana™ Isolation Kit (Ambion, USA) following the manufacturer’s instructions. RNA was isolated in four biological replicates, each consisting of 20 embryos. In total, 32 RNA samples were extracted. RNA concentration and quality were assessed using a NanoDrop spectrophotometer (NanoDrop Technologies, Wilmington, USA) and Agilent Bioanalyzer (Agilent Technologies, Santa Clara, USA).

### Library construction and Illumina sequencing

In total, 32 RNA samples (30 µL each) obtained from the germinating embryos at 1 DAI of four tested barley genotypes ('Sebastian' (wild-type), *hvcbp20.ab*, *hvcbp80.b*, *hvcbp20.ab/hvcbp80.b*), each in four biological replicates, subjected to two treatment conditions (control and 75 µM ABA) at 1DAI, were used for TruSeq stranded mRNA cDNA library construction and next-generation sequencing (NGS) (Macrogen Inc., South Korea). Illumina RNA-seq was performed using a NovaSeq6000 sequencer (40 M, 2xPE 150 bp). Preprocessing of the raw reads was performed using FastQC, adapters were removed with Cutadapt, and quality control of the trimmed reads was checked again with FastQC^97^. Low-quality reads were filtered using Cutadapt software. Paired-end reads were mapped to the BaRTv2.18^51^ barley reference transcriptome using the Kallisto tool^98^. The mapped reads were quantified and normalized to transcripts per million (TPM) using Kallisto.

### Differential expression analysis

Differential gene and transcript expression analyses were performed using the limma-voom package in a 3D RNA-seq application^99^. Analysis was performed for two treatment groups (ABA and control) and seven comparisons (contrast groups): under control conditions between the *hvcbp20.ab*, *hvcbp80.b*, *hvcbp20.ab/hvcbp80.b* mutants against the 'Sebastian' parental variety (control.cbp20-control.WT, control.cbp80-control.WT, control.double-control.WT) and after ABA treatment of each genotype (*hvcbp20.ab*, *hvcbp80.b*, *hvcbp20.ab/hvcbp80.b* and wild type 'Sebastian') against control conditions (ABA.WT-control.WT, ABA.cbp20-control.cbp20, ABA.cbp80-control.cbp80, ABA.double-control.double). In all contrast groups, differentially expressed genes (DEG), which show significant changes in expression levels between tested conditions; differentially expressed transcripts (DET), specific transcripts of genes exhibiting significant expression changes compared to other transcripts within the same gene; differential alternative splicing (DAS), where genes with multiple transcript isoforms show varying abundance patterns between conditions; and differential transcript usage (DTU), highlighting specific transcript variants preferentially expressed between tested conditions, were analyzed. The threshold for significant differential gene and transcript expression was log_2_FC ≥ 1.5 or ≤ -1.5, with a Benjamini-Hochberg (BH) adjusted p-value < 0.01. To identify significant DAS, the percentage spliced (ΔPS) threshold was set at 0.5. The DAS genes within each contrast group were identified based on an adjusted p-value below the predefined threshold (< 0.01) and the presence of at least one transcript exhibiting a ΔPS greater than 0.5. For DTU identification, each transcript's expression was compared to the weighted average expression of all other transcripts within the same gene. A transcript was classified as DTU if its adjusted p-value was below the predefined threshold (< 0.01) and the absolute value of ΔPS exceeded the defined cut-off value of 0.5. To identify common DEGs and DETs between all tested genotypes, and exclusive for each genotype in the treatment groups, Venn diagrams were constructed using the Venny 2.1 tool (https://bioinfogp.cnb.csic.es/tools/venny/). In this study, we focused on the genes and transcripts specific for *hvcbp20.ab/hvcbp80.b*. Gene Ontology enrichment analysis was performed using TopGO (version 2.50.0) implemented in R (version 4.2.1) with an adjusted p-value < 0.01. The BH method was used to correct the False Discovery Rate (FDR).

### Transcription factors (TFs) identification and binding site prediction

The PlantRegMap tool was used to predict transcription factors (TFs)^100^. Promoter sequences, defined as the 1500 bp upstream flanking regions, were retrieved through EnsemblPlants v. 45 using the BioMart tool, based on the MorexV3 barley genome version integrated into the EnsemblPlants database^101^. These promoter sequences were analyzed for transcription factor-binding sites (TFBS) using the PlantRegMap Binding Site Prediction feature. Subsequently, datasets detailing TFs and their potential target genes were merged to explore the potential regulatory interactions.

### Identification of Arabidopsis homologs

The BLAST tool within the Ensembl Plants database was used to identify Arabidopsis homologs (version of the genome TAIR10). A threshold of at least 40% similarity between sequences was set for homology^102^.

### Analysis of interactome

Using the STRING database (version 11.5), a predicted network of physical protein–protein interactions (PPIs) was constructed for the DEGs and DAS genes in the *hvcbp20.ab/hvcbp80.b* mutant in ABA presence. For network analysis, the physical subnetwork was selected, and the default parameters were used with a medium confidence interaction score threshold of 0.4 and no additional interactors. The PPi network was visualized using Cytoscape software^103^.

### Co-expressed genes analysis

All significantly differentially expressed genes (DEGs) identified within the RNA-seq experiment of germinating embryos at 1 DAI from tested mutants and WT were used to identify gene co-expression clusters. The average transcript per million (TPM) gene values of four biological replicates of the ABA-treated samples were used in the 'clust 1.10.8' tool in Linux with default settings was employed for this purpose. Heatmaps were generated using the R package 'Pheatmap' v.1.0.12, based on TPM values.

### Prediction of splicing factors

Splicing factors (SF) were searched by filtering the gene ontology (GO) term ‘RNA splicing’ (GO:0008380) from a list of exclusively differentially expressed genes (DEG) and a list of exclusively differentially expressed transcripts (DET) in each genotype studied under ABA vs. control conditions.

### Metabolome analysis

Embryos extracted from germinating seeds were homogenized in liquid nitrogen and subsequently chilled on ice. Precise 40 ± 1 mg of this tissue was then transferred into microcentrifuge tubes, followed by the addition of 1 mL of chloroform: methanol: dH_2_O mixture (1:2.5:1). The samples were vigorously mixed using by vortexing at 4 °C for 15 min before being placed back on ice. Afterward, they were centrifuged at 4 °C at 5000×*g* for 3 min. The supernatants, which contained both polar and nonpolar metabolites, were carefully transferred to new tubes and dried using a Buchi Rotavapor system at 25 °C, to avoid complete evaporation. Finally, 100 µL of the supernatant was reserved for metabolomic profiling using liquid chromatography-tandem mass spectrometry (LC–MS/MS) as described by Baptista et al.^104^.

### PacBio sequencing and reference transcriptome dataset (RTD)

Eight RNA SMRTbell Iso-seq libraries were prepared using RNA extracted from the same samples as those used for Illumina sequencing but pooled for each genotype. Further libraries were sequenced using the PacBio sequel II platform according to the manufacturer’s protocol (Macrogen Inc., Seoul, Republic of Korea). To investigate whether transcriptomic analysis specific to the Sebastian genotype was overlooked using BaRTv2.18, a Barke-based transcriptome, and assess the potential necessity of assembling a Sebastian-specific reference transcript dataset (RTD) in future endeavors, we adhered to the BaRTv2.18 assembly pipelines, assembling RTDs from both Illumina RNA-seq short reads and PacBio Iso-seq long reads^51^. Specifically, the STAR mapping tool^105^ was used to align the trimmed RNA-seq reads of the 32 samples to the Barke reference genome^106^. Transcript models were assembled from the read alignment using two assemblers: Stringtie^107^ and Scallop^108^. The resultant transcripts in different samples, along with those from BaRTv2.18, were merged to generate an RNAseq RTD using RTDmaker (https://github.com/anonconda/RTDmaker). In addition, PacBio Iso-seq data were generated from four samples (a pool of RNA was used for this purpose per genotype analyzed). After pre-processing the Iso-seq data with Isoseq3 pipelines (https://github.com/PacificBiosciences/IsoSeq), full-length non-concatemer (FLNC) reads were mapped to the Barke reference genome using Minimap2^109^. Transcript models were assembled through TAMA, and transcript start and end sites, as well as splice junctions, were refined using the BaRTv2.18 approach^51^, yielding Isoseq RTD. Leveraging the full-length transcript capability of PacBio, we integrated transcripts from the RNA-seq RTD that contributed to novel splice junctions or gene loci into the Isoseq RTD, resulting in a more comprehensive BarkeRTD. Comparative analyses of splice junctions and transcript-level intron combinations across BarkeRTD, Isoseq RTD, RNA-seq RTD, and BaRTv2.18, were conducted to ascertain whether BaRTv2.18 may have overlooked critical information about spliced transcripts.

### Statement regarding experimental research on plants

Experimental research on plants complied relevant institutional, national, and international guidelines and legislation.

### Supplementary Information

Supplementary Information 1.

Supplementary Information 2.

## Data Availability

The data generated and analyzed in this research article are included in all figures, tables, and supplemental data. The RNA-seq data used in the present study have been deposited into EMBL-EBI (EMBL’s European Bioinformatics Institute) in the Array Express repository (https://www.ebi.ac.uk/) under accession number E-MTAB-13989.
