# Supplementary material for: The cap-binding complex modulates ABA-responsive transcript splicing during germination in barley (Hordeum vulgare)
Source: Sci Rep. 2024 Aug 7;14:18278. doi: 10.1038/s41598-024-69373-9 (PMC11303550; doi:10.1038/s41598-024-69373-9)
Supplement: Supplementary file 1 — Supplementary Information 1. [file 41598_2024_69373_MOESM1_ESM.docx]

**Article title:** The Cap-Binding Complex Modulates ABA-Responsive Transcript Splicing During Germination in Barley (Hordeum vulgare)

**Authors:** Ewa Sybilska^1^, Anna Collin^2^, Bahar Haddadi^2^, Luis Mur^2^, Manfred Beckmann^2^, Wenbin Guo^3^, Craig G. Simpson^4^, Agata Daszkowska-Golec^1*^

The following Supporting Information is available for this article:

**Supplementary Table 1** Exclusively differentially expressed transcription factors in *hvcbp20.ab/hvcbp80.b* after 75 µM ABA treatment compared to control conditions.

| **TF family** | **Description** | **No.** | **Arabidopsis homolog** | **Gene ID** |
| --- | --- | --- | --- | --- |
| MYB | MYB domain protein | 2 | MYB33 | AT5G06100 |
|  |  |  | CDC5 | AT1G09770 |
| MYB_related | MYB-related protein | 3 | MYBD | AT1G70000 |
|  |  |  | MYB16 | AT5G15310 |
|  |  |  | N/A | AT5G56840 |
| WRKY | WRKY family transcription factor | 2 | WRKY11 | AT4G31550 |
|  |  |  | WRKY13 | AT4G39410 |
| NAC | NAC domain-containing protein | 2 | NAC066 | AT3G61910 |
|  |  |  | NAC016 | AT1G34180 |
| bZIP | Basic-leucine zipper protein | 3 | TGA6 | AT3G12250 |
|  |  |  | TGA9 | AT1G08320 |
|  |  |  | TGA10 | AT5G06839 |
| C2H2 | C2H2-type zinc finger family protein | 3 | NUC | AT5G44160 |
|  |  |  | TFIIIA | AT1G72050 |
|  |  |  | MGP | AT1G03840 |
| C3H | Zinc finger (CCCH-type) family protein | 1 | HUA1 | AT3G12680 |
| bHLH | Basic helix-loop-helix protein | 2 | SRS8 | AT5G33210 |
|  |  |  | LRL1 | AT2G24260 |
| ERF | Ethylene responsive transcription factor | 2 | Rap2.6L | AT5G13330 |
|  |  |  | ERF35 | AT3G60490 |
| HB-other | Homeobox (HB)-other family protein | 2 | SHH2 | AT3G18380 |
|  |  |  | LD | AT4G02560 |
| CPP | Cystein-rich polycomb-like protein | 1 | TCX8 | AT3G16160 |
| E2F/DP | E2F/DP family protein | 1 | DPA | AT5G02470 |
| Total TFs |  | 24 |  |  |

**Supplementary Table 2** Basic statistics of BarkeRTD, Isoseq RTD, RNAseq RTD and BaRTv2.18.

| **Stats** | **BarkeRTD** | **Isoseq** | **RNAseq** | **BaRTv2.18** |
| --- | --- | --- | --- | --- |
| Genome covered bases | 88,058,611 | 37,259,798 | 95,050,413 | 88,172,513 |
| Gene number | 44,137 | 20,020 | 41,410 | 39,434 |
| Multi-isoform gene number | 22,239 | 13,554 | 19,514 | 20,230 |
| Mono-exon gene number | 14,363 | 3,014 | 14,242 | 11,961 |
| Multi-exon gene number | 29,774 | 17,006 | 27,168 | 27,473 |
| Transcript number | 157,233 | 69,225 | 158,026 | 148,260 |
| Mono-exon transcript number | 22,954 | 10,212 | 17,842 | 17,411 |
| Multi-exon transcript number | 134,279 | 59,013 | 140,184 | 130,849 |
| Transcript number per gene | 3.562 | 3.458 | 3.816 | 3.76 |
| Exon number | 1,043,178 | 414,887 | 1,109,872 | 1,019,852 |
| Exon number per transcript | 6.635 | 5.993 | 7.023 | 6.879 |
| Exon average length | 304.1797 | 283.2127 | 313.8507 | 310.7342 |
| Intron number | 885,945 | 345,662 | 951,846 | 871,592 |
| Intron number per transcript | 5.635 | 4.993 | 6.023 | 5.879 |
| Intron average length | 546.0459 | 410.0681 | 526.6979 | 525.4366 |
| Transcript N50 | 2,247 | 1,847 | 2,457 | 2,382 |
| Transcript N90 | 1,223 | 1,131 | 1,367 | 1,317 |
| Transcript average length (exonic) | 2,018.11 | 1,697.38 | 2,204.28 | 2,137.48 |
| Transcript total length (exonic) | 317,313,620 | 117,501,261 | 348,334,062 | 316,902,925 |


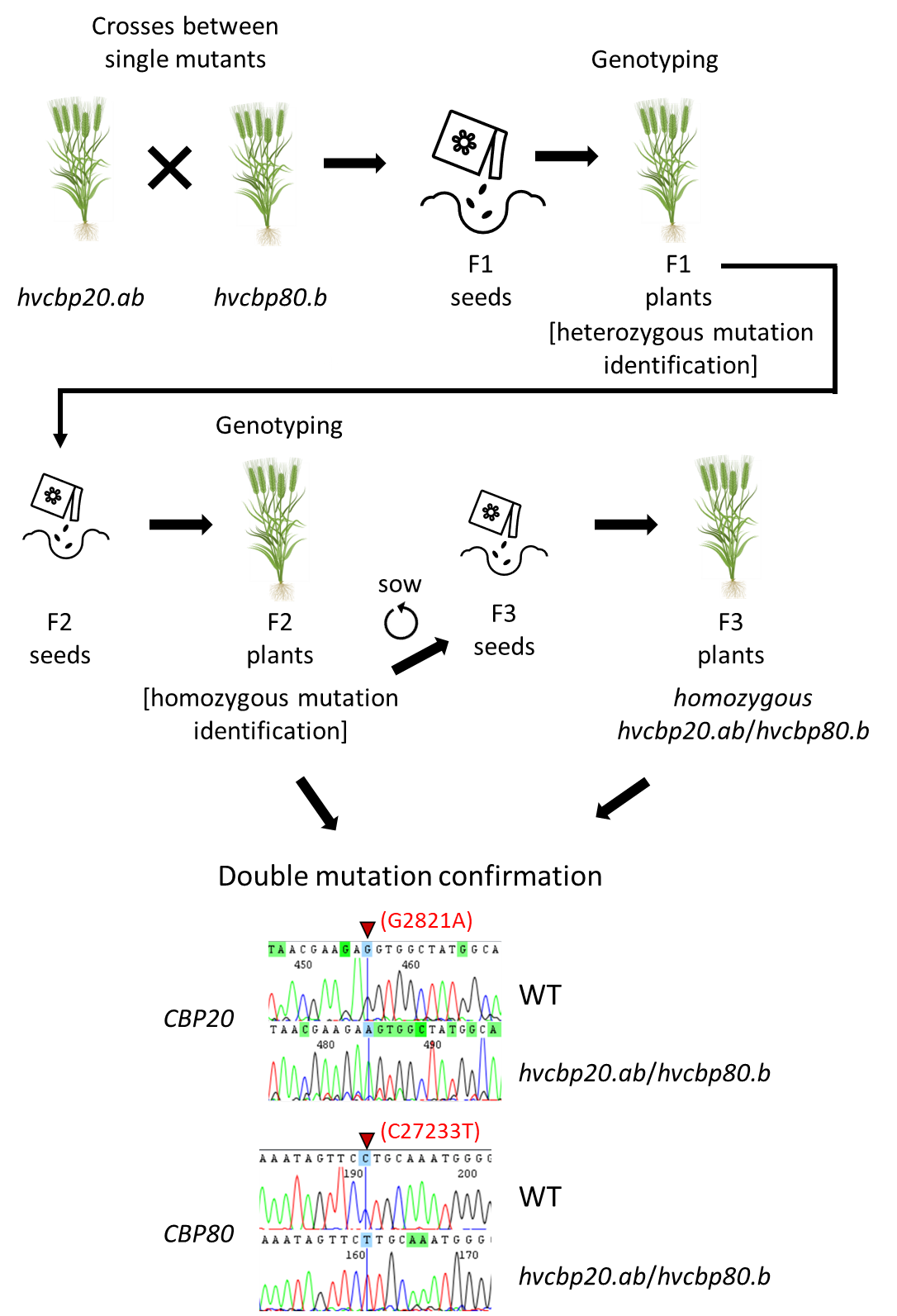


**Supplementary Fig. S1** Schematic diagram of *hvcbp20.ab/hvcbp80.b* generation. Position of mutation based on HORVU.MOREX.r3.2HG0179950 and HORVU.MOREX.r3.4HG0377830 (respectively CBP20 and CBP80). Mutation in the *CBP20* and *CBP80* genes in *hvcbp20.ab/hvcbp80.b* double mutant were confirmed using Sanger sequencing.


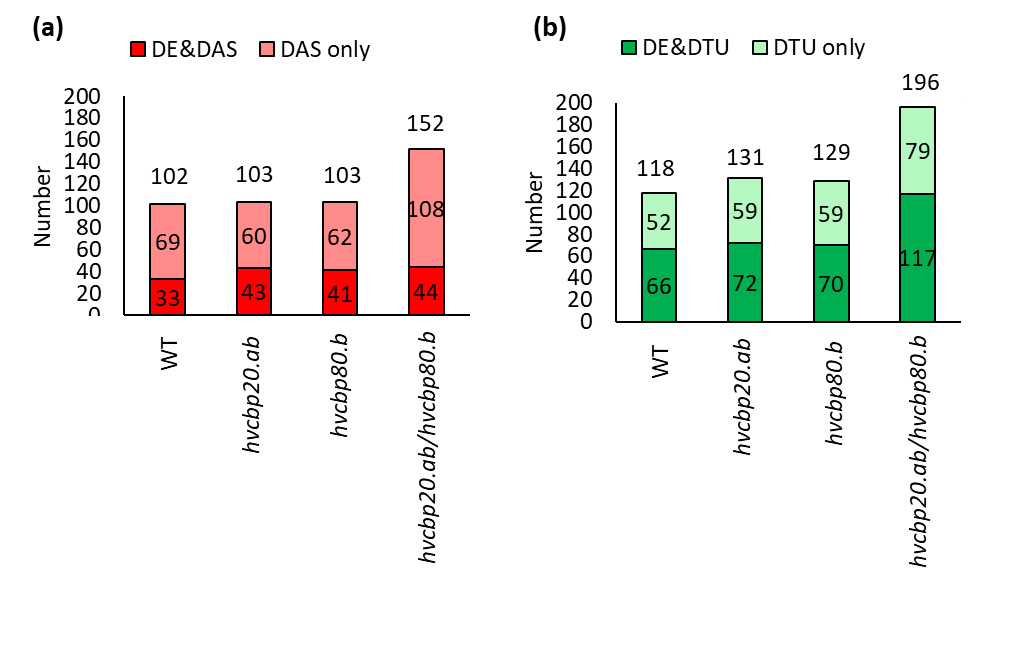


**Supplementary Fig. S2** Analysis of the number of genes with differential alternative splicing (DAS) and transcripts with differential transcript usages (DTU) in *hvcbp20.ab*, *hvcbp80.b*, *hvcbp20.ab/hvcbp80.b* and WT after 75 µM ABA treatment compared to control conditions. **(A)** The number of DE&DAS genes and DAS only genes. **(B)** The number of DE&DTU transcripts and DTU only transcripts. DE (differential expression),, DAS (differential alternative splicing) genes, DTU (differential transcript usage) transcripts.


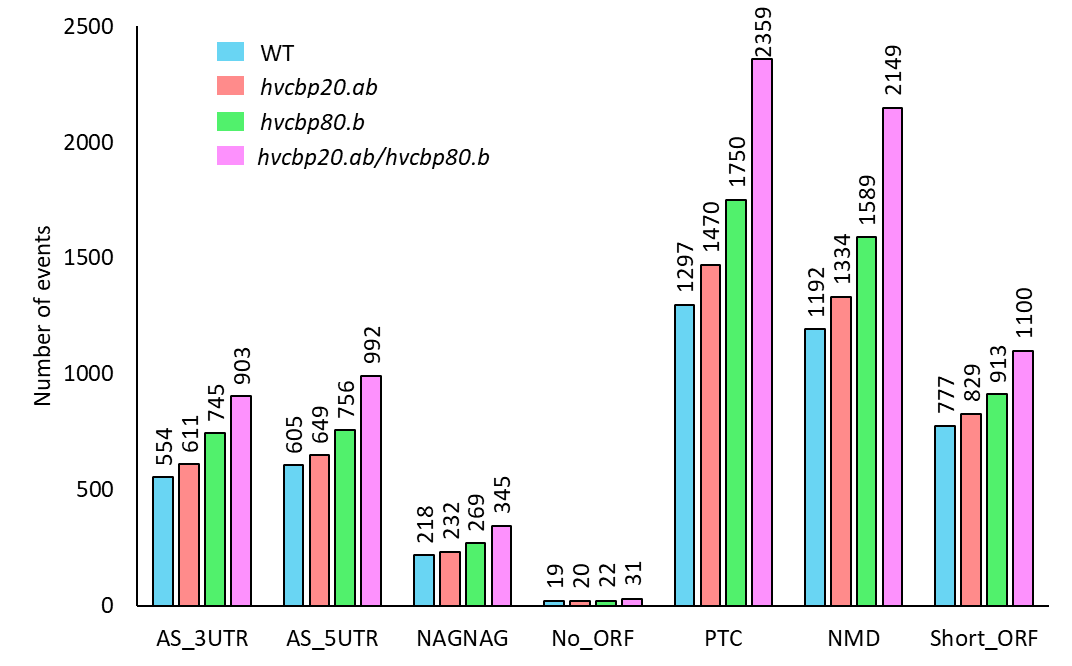


**Supplementary Fig. S3** Features of differentially expressed transcripts (DET) in *hvcbp20.ab*, *hvcbp80.b*, *hvcbp20.ab/hvcbp80.b* and WT after 75 µM ABA treatment compared to control conditions. alternative splicing in 3′ untranslated region (AS_3′UTR), alternative splicing in 5′ untranslated region (AS_5′UTR), tandem acceptor sites (NAGNAG), no open reading frame (No_ORF), premature termination codon (PTC), nonsense-mediated mRNA Decay (NMD), short open reading frame (Short_ORF).

**Supplementary Data S1 – S14 (see separate Excel file)**

**Supplementary Data S1.** List of co-expressed genes within C0-C11 clusters in *hvcbp20.ab*, *hvcbp80.b*, *hvcbp20.ab/hvcbp80.b* and wild-type 'Sebastian' in the presence of 75 µM ABA.

**Supplementary Data S2.** GO biological process analysis of exclusively differentially upregulated /downregulated genes in *hvcbp20.ab/hvcbp80.b* in the presence of 75 µM ABA compared to control conditions.

**Supplementary Data S3.** List of genes in top 5 overrepresented GO biological process of exclusively differentially upregulated / downregulated genes in *hvcbp20.ab/hvcbp80.b* in the presence of 75 µM ABA compared to control conditions.

**Supplementary Data S4.** Splicing factors among exclusively differentially expressed genes (DEG) in *hvcbp20.ab/hvcbp80.b* in the presence of 75 µM ABA compared to control conditions.

**Supplementary Data S5.** Genes related to negative brassinosteroid signaling pathway among exclusively differentially expressed genes (DEG) in *hvcbp20.ab/hvcbp80.b* in the presence of 75 µM ABA compared to control conditions.

**Supplementary Data S6.** List of the exclusively differentially expressed transcription factors detected after 75 µM ABA treatment compared to control conditions in each contrast group. **Supplementary Data S7.** List of exclusively differentially expressed transcription factors with binding sites among exclusively differentially expressed genes in *hvcbp20.ab/hvcbp80.b* in the presence of 75 µM ABA compared to control conditions.

**Supplementary Data S8.** List of exclusively differentially expressed transcription factors with binding sites among exclusively differentially expressed genes encoding splicing factors in *hvcbp20.ab/hvcbp80.b* in the presence of 75 µM ABA compared to control conditions.

**Supplementary Data S9.** List of exclusive genes with differential alternative splicing (DAS) in the presence of 75 µM ABA compared to control conditions in each contrast group.

**Supplementary Data S10.** List of exclusive differential transcript usages (DTU) in the presence of 75 µM ABA compared to control conditions in each contrast group.

**Supplementary Data S11.** GO biological process analysis of genes of exclusively differentially upregulated/downregulated transcripts in *hvcbp20.ab/hvcbp80.b* in the presence of 75 µM ABA compared to control conditions.

**Supplementary Data S12.** List of the exclusively differentially expressed transcripts (DET) of splicing factors in the presence of 75 µM ABA compared to control conditions in each contrast group.

**Supplementary Data S13.** Mapping of the protein-protein interactome predictions among CBP20 and CBP80 subunits in *hvcbp20.ab/hvcbp80.b*.

**Supplementary Data S14.** List of differentially expressed genes (DEG) and genes with differential alternative splicing (DAS) encoding proteins that in silico physically interact with CBP20 and CBP80 subunits in *hvcbp20.ab/hvcbp80.b* in the presence of 75 µM ABA compared to control conditions.
